# Cholesterol in Relation to COVID-19: Should We Care about It?

**DOI:** 10.3390/jcm9061909

**Published:** 2020-06-18

**Authors:** Dina Radenkovic, Shreya Chawla, Matteo Pirro, Amirhossein Sahebkar, Maciej Banach

**Affiliations:** 1 Guy’s and St Thomas’ Hospital, London SE1 7EH, UK; dina.radenkovic@kcl.ac.uk; 2 Faculty of Life Sciences and Medicine, King’s College London, London SE5 9NU, UK; shreya.chawla@kcl.ac.uk; 3 Unit of Internal Medicine, Angiology and Arteriosclerosis Diseases, Department of Medicine, University of Perugia, 06123 Perugia, Italy; matteo.pirro@unipg.it; 4 Halal Research Center of IRI, FDA, Tehran 314715311, Iran; amir_saheb2000@yahoo.com; 5 Biotechnology Research Center, Pharmaceutical Technology Institute, Mashhad University of Medical Sciences, Mashhad 9177948564, Iran; 6 Neurogenic Inflammation Research Center, Mashhad University of Medical Sciences, Mashhad 9177948564, Iran; 7 Department of Hypertension, WAM University Hospital in Lodz, Medical University of Lodz (MUL), Zeromskiego 113, 90-549 Lodz, Poland; 8 Polish Mother’s Memorial Hospital Research Institute (PMMHRI), 93-338 Lodz, Poland; 9 Cardiovascular Research Centre, University of Zielona Gora, 65-417 Zielona Gora, Poland

**Keywords:** atherosclerosis, cholesterol, coronavirus, COVID-19, lipid-lowering therapy, SARS-CoV-2, statins

## Abstract

Current data suggest that infection with severe acute respiratory syndrome coronavirus 2 (SARS-CoV-2) causing corona virus disease-19 (COVID-19) seems to follow a more severe clinical course in patients with cardiovascular disease (CVD), hypertension, and overweight/obesity. It appears that lipid-lowering pharmacological interventions, in particular statins, might reduce the risk of cardiovascular complications caused by COVID-19 and might potentially have an additional antiviral activity. It has been shown that high cholesterol levels are associated with more lipid rafts, subdomains of the plasma membrane that can harbour angiotensin-converting enzyme 2 (ACE2) receptors for the S-protein of SARS-CoV-2. Evidence of the importance of cholesterol for viral entry into host cells could suggest a role for cholesterol-lowering therapies in reducing viral infectivity. In addition to their lipid-lowering and plaque-stabilisation effects, statins possess pleiotropic effects including anti-inflammatory, immunomodulatory, and antithrombotic activities. Lower rates of mortality and intubation have been reported in studies investigating statin therapy in influenza infection, and statin therapy was shown to increase viral clearance from the blood during chronic hepatitis C infection. Statins may also serve as potential SARS-CoV-2 main protease inhibitors, thereby contributing to the control of viral infection. In this review, we elaborate on the role of cholesterol level in the process of the coronavirus infection and provide a critical appraisal on the potential of statins in reducing the severity, duration, and complications of COVID-19.

## 1. Introduction

Severe acute respiratory syndrome coronavirus 2 (SARS-CoV-2), responsible for the current pandemic of corona virus disease-19 (COVID-19), represents a great challenge to healthcare systems worldwide. At present, indisputably effective drug therapies against SARS-CoV-2 are still not available [1]; thus, the most effective preventive strategy is to avoid being exposed to the virus [2]. 

Observational studies of COVID-19 individuals with underlying cardiovascular disease (CVD) showed that they were at increased risk of severe manifestations of the disease and mortality [3]. Wang et al. found that SARS-CoV-2 patients needing intensive care unit (ICU) admission were older and more likely to having underlying comorbidities, including hypertension (58.3% vs. 21.6%), diabetes (22.2% vs. 5.9%), CVD (25% vs. 10.8%), and cerebrovascular disease (16.7% vs. 1%) compared to those who did not need ICU treatment [4]. Furthermore, the Chinese Centre for Disease Control and Prevention published a case series of COVID-19, with an overall case fatality rate of 2.3%. Importantly, in patients with underlying CVD, the mortality rate was 10.5% (in comparison to 7.3% for diabetes, 6.3% for chronic respiratory disease, 6.0% for hypertension, and 5.6% for cancer) [5]. A subsequent study reported similar findings [6]. Patients with high troponin T (TnT) levels (in response to SARS-Cov-2 infection) were more likely to develop COVID-19 complications, including acute respiratory distress syndrome (ARDS) (57.7% vs. 11.9%), malignant arrhythmias (17.3% vs. 1.5%), acute coagulopathy (65.8% vs. 20.0%), and acute kidney injury (36.8% vs. 4.7%), as compared with those with normal TnT levels. Patients with underlying CVD were more likely to exhibit high levels of TnT (54.5% vs. 13.2%) and had a nine times greater mortality rate than those with normal TnT levels and without CVD (69.44 vs. 7.62%) [6]. All these findings suggest that there is a significant predisposition to COVID-19 complications and mortality in patients with CVD. Therefore, CVD prevention strategies are extremely important in the COVID-19 pandemic, especially strategies with a wide spectrum of possible beneficial effects. It appears that statins may be especially useful, as they not only reduce the risk of cardiovascular complications, but may have independent antiviral, anti-inflammatory, and antithrombotic effects [7,8,9]. The combination of these effects may translate into an overall health benefit in COVID-19 patients. 

This paper summarises the most relevant data on the interaction of CVD risk factors and treatment options with COVID-19, with a particular focus on the role of cholesterol and cholesterol-lowering therapy.

## 2. Lipid Rafts, Cholesterol, and Viral Entry 

**Figure 1:**
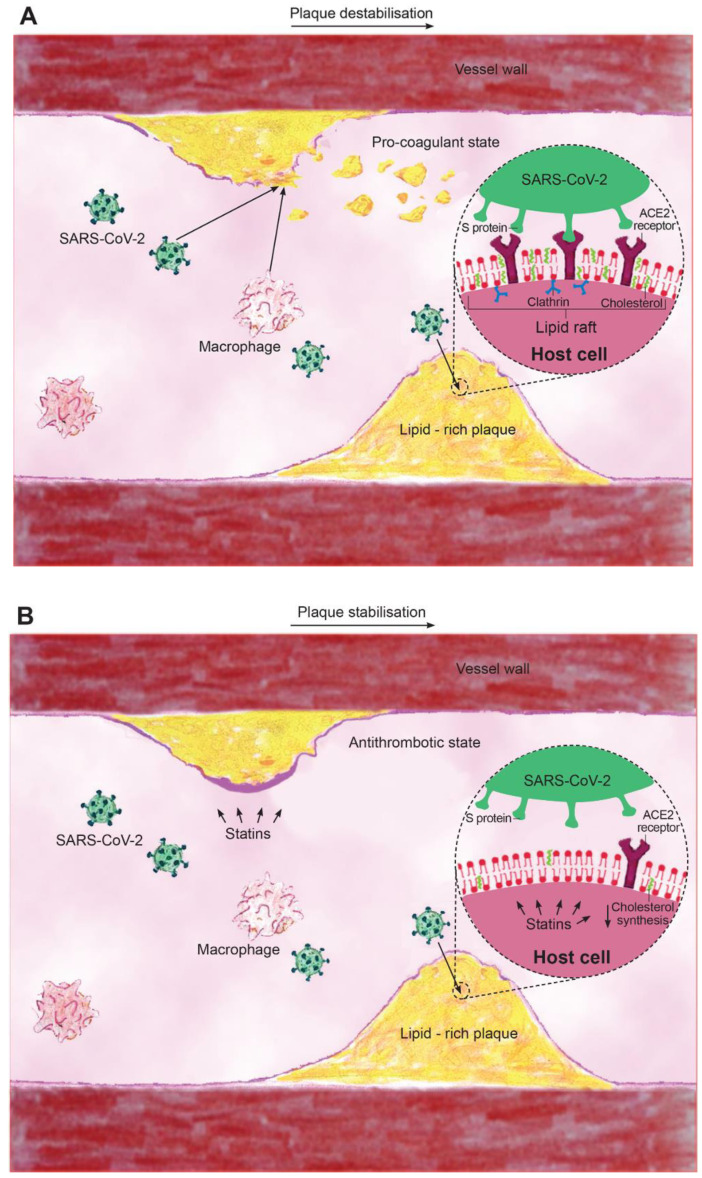
Proposed mechanism of the role of cholesterol and statins in severe acute respiratory syndrome coronavirus 2 (SARS-CoV-2) infection. (**A**) Lipid rafts rich in cholesterol serve as docking sites for angiotensin-converting enzyme 2 (ACE2) receptors and viral attachment via the S protein of SARS-CoV-2, which is then taken into the cells by clathrin. In addition, acute infection with SARS-COV-2 and macrophages via paracrine factors can lead to plaque instability and embolization causing occlusion of distal microvasculature. (**B**) Statins disrupt lipid rafts and viral binding; reduce cholesterol; and have plaque stabilising, antithrombotic, and anti-inflammatory properties.

In order to further characterise the underlying mechanisms of SARS-CoV-2 and CVD, it is important to understand the interaction of the virus with the host cell. Coronavirus is a single-stranded, positive sense RNA virus with a lipid envelope. The virus has four structural proteins: the nucleocapsid protein, membrane protein, envelope protein, and the spike protein (S glycoprotein), which mediates attachment to the angiotensin converting enzyme 2 (ACE2) receptor [10,11]. Lipid rafts are subdomains of the plasma membrane enriched in cholesterol and glycosphingolipids, which have been shown to play an important role in viral entry into host cells [12,13]. The abundant presence of cholesterol within lipid rafts is thought to play an essential role in promoting viral infectivity [12,13]. Lipid rafts are important for the interaction between the S protein and ACE2 receptor as well as for facilitating the process of viral endocytosis [14]. However, the localisation of ACE2 on lipid rafts has been a topic of controversy [13,14,15,16,17], e.g., Lu et al. [14] showed that ACE2 was largely co-localized with the raft marker caveolin-1 and GM1, and that ACE2 was shifted to the non-raft environment after depletion of cholesterol. In addition, lipid rafts also contain caveolins, clathrins, and dynamin, which may be as important as cholesterol in the process of viral entry [11] (Figure 1A). 

The role of cholesterol in viral entry has been studied for several coronaviruses including SARS-CoV [14], murine coronavirus [15], porcine deltacoronavirus [12], and infectious bronchitis virus [16]. Thus, cholesterol present in the cell membrane and viral envelope has been found to contribute to coronavirus replication by acting as a key component in viral entry [3,11]. Moreover, cholesterol was recently shown to be involved in binding and altering the oligomeric status of the N-terminal fusion peptide of SARS-CoV, which is essential for virus entry into the host cell [18]. The impact of cholesterol on coronavirus infectivity was further supported by examining the effect of depleting cholesterol on SARS-CoV infection, which resulted in a significant reduction in viral mRNA [14]. It was observed that cholesterol depletion impaired viral entry and virus-induced fusion, suggesting that cholesterol is important during the post-binding stages [13]. Although the above reported in vitro data suggest an essential role of lipid rafts and cholesterol in viral entry, specific confirmation in vivo is needed. The seemingly important role of lipid rafts in promoting cellular entry of coronavirus can guide new therapies that may be directed against SARS-CoV-2. 

## 3. Cholesterol-Lowering, Cardiovascular Complications of Acute Respiratory Viruses

In addition to the importance of cardiovascular risk factors in exacerbating acute respiratory infections [3,10], there is also evidence showing that viral infections may cause cardiac complications [6]. Accordingly, previous studies have established acute respiratory viral infections such as those caused by influenza virus and coronaviruses to trigger cardiovascular (CV) complications such as acute coronary syndrome (ACS) [19], myocarditis, arrhythmias [20], and heart failure (HF) acceleration. There have been several reports on the burden of myocardial injury in the COVID-19 pandemic [6,21,22,23,24]. Huang et al. [21] identified virus-related cardiac injury in 12% of patients who had raised troponin I. Similarly, in three independent studies, evidence of cardiac injury was found in 7.2, 19.7 [22], and 29% of COVID-19 patients [23], respectively. Also, Guo et al. identified 27.8% of COVID-19 patients as exhibiting myocardial injury as indicated by elevated TnT [6]. Conversely, patients with myocardial injury showed significantly higher mortality than those with normal TnT levels (59.6% vs. 8.9%) [6]. Similarly, Shi et al. reported a higher mortality rate in those with vs. without cardiac injury (51.2% vs. 2.4%) [22]. Those who developed myocardial injury had a more severe clinical course with higher reports of needing to be assisted with ventilation and also with a higher mortality [6,22,25]. Several mechanisms have been suggested for the increase in cardiac injury seen in COVID-19 patients. Some authors have suggested that the cytokine storm caused by COVID-19 may result in the development of fulminant myocarditis [6,10,21]. Such mechanisms have also been reported in previous viral epidemics such as SARS-CoV and Middle East respiratory syndrome (MERS), which have been characterised by cytokine storms resulting in their highly pathological effects on lungs and other organs [26]. Shi et al. further proposed that the acute inflammatory response that has been reported in COVID-19 might exacerbate the inflammatory activity within atherosclerotic plaques as well as cause endothelial dysfunction, finally resulting in atherothrombotic complications. Thrombo-inflammation can further exacerbate cardiac ischemia and injury [22]. An additional mechanism possibly explaining the detrimental impact of COVID-19 on CV complications includes the affinity of SARS-COV-2 for the ACE2 receptor, which is highly expressed in the heart [3,6,22], suggesting the possibility of direct viral infection of the heart. This hypothesis is further supported by previous reports detecting the presence of the viral genome in 35% of SARS-CoV infected autopsied hearts [6,27], suggesting the potential for direct myocardial damage.

The apparent effects of COVID-19 at exacerbating cardiac injury, inflammation, and plaque activity have resulted in the hypotheses that drugs reducing all these unfavourable outcomes might be effective resources for COVID-19 management [3]. In this context, the pleiotropic effects of statins (beneficial effects beyond cholesterol-lowering) include anti-inflammatory, immunomodulatory, and antithrombotic properties, which, importantly, can be observed before lipid-lowering is evident [7,8,9,28,29]. The inflammation, and especially cytokine storm, caused by COVID-19 could be expected to result in greater plaque instability and more extensive remodelling [30]—fundamental morphologies of high-risk plaques [31]. It has been shown that statin therapy promotes regression and stabilisation of vulnerable plaques, and the effect seems to be dose dependent [32]. Statins may also significantly reduce regional arterial wall inflammation [33]. This suggests that COVID-19 patients with baseline CVD, being at very high or extremely high cardiovascular risk, require optimal intensive statin therapy with maximally tolerated doses in order to promote plaque stability [34] (Figure 1B). 

In addition to its cholesterol-lowering and plaque-stabilisation effects, the pleiotropic effects of statins include attenuation of chronic low-grade inflammation [35] and the immune response to infection. These effects may be beneficial in the context of the cytokine storm caused by COVID-19 [6,21]. Their anti-inflammatory effects have been postulated to be a result of reduction of isoprenylation, which reduces the magnitude of the signalling pathways and thus counteracts the cytokine storm [28,36]. This suggests statins may have the potential to significantly reduce the inflammatory burden and exacerbation of the clinical course of COVID-19, which might otherwise result in ARDS and myocarditis. These effects might also be the result of the reported antiviral effects of statins (Figure 1B). 

## 4. Use of Statins in Human Viral Infections

Although there has been almost no evidence on the use of statins in patients with SARS-CoV-2, these drugs have previously been investigated in the treatment of other acute respiratory viral infections such as influenza [37,38,39]. A study of 1055 adult patients with viral pneumonia found lower rates of mortality and intubation with continued use of statins throughout the hospital stay (odds ratio (OR) 0.26; 95% confidence interval (CI): 0.08–0.81) [37]. Similar to SARS-CoV, hepatitis C viral (HCV) replication is closely associated with lipid metabolism, and statins are expected to disrupt this mechanism [40]. Statin treatment in chronic HCV was shown to increase the clearance of the virus from the blood, down-regulate HCV replication [41], and resulted in clinical reduction in hepatocellular carcinoma [41,42]. Additionally, a meta-analysis by Chopra et al. showed that statin use was associated with lower mortality after pneumonia (OR 0.62, 95% CI: 0.54–0.71) [43]. 

A significant complication of the COVID-19 infection is the development of ARDS. Approximately 5% of COVID-19 patients will require intensive care [44] and mechanical ventilation. Makris et al. [45] investigated the impact of pravastatin therapy on ventilator-associated pneumonia (VAP) frequency and mortality. The authors showed that the pravastatin group had significantly increased probability of survival compared to controls during the 30-day treatment period (*p* = 0.04), additionally, VAP frequency was reduced (25.3% vs. 38.2%) [45]. Conversely, another analysis found no benefit of statin administration on day-28 mortality in patients with VAP [46]. However, this study was limited by a short duration of simvastatin administration, which was probably administered too late to see an effect—patients received a statin after several days in the ICU and had dysfunction of at least one organ [46]. Other studies focused on statin therapy to prevent sepsis or against community-acquired infections [37,38,47] supported a role for these drugs in severe infections. 

Nevertheless, total cholesterol levels in admitted COVID-19 patients can be extremely variable and time-dependent. A recent study revealed that COVID-19 patients had sharply decreased total cholesterol and low-density lipoprotein cholesterol (LDL-C) levels (3.70 ± 0.09 mmol/L (143 ± 3.5 mg/dL), and 1.82 ± 0.08 mmol/L (70.4 ± 3.1 mg/dL), respectively, *p* < 0.001 for both) [48]. Although several mechanisms for the acute fall in cholesterol were suggested, it still remains unclear whether these changes in serum cholesterol are related to viral–host cell fusion and entry [48], thus, the timing of cholesterol lowering may be fundamental in the management of critically unwell patients, and these therapies might be better suited earlier in the disease course prior to critical care admission. Lastly, a recent in silico analysis showed that several statins could serve as potential SARS-CoV-2 main protease inhibitors, with pitavastatin, a highly lipophilic molecule, exhibiting the strongest binding [49]. There are also some reports suggesting that statins might enhance ACE2, which might mitigate the invasion of SARS-CoV-2 through the ACE2 receptor [50]. All these results seem encouraging but need to be confirmed in further observational and interventional clinical studies. 

## 5. Future Perspective 

Further investigation is needed on the role of cholesterol and use of statins amongst patients with COVID-19 infections. The safety and availability of statins makes it worthwhile to consider whether such host-response modulating drugs may promote a milder clinical infection if initiated early in the disease process. We also should keep in mind the possible occurrence of muscle symptoms during the course of COVID-19. While myalgias are easily attributable to SARS-CoV-2 infection in statin untreated patients, their differential diagnosis may be cumbersome in COVID-19 patients receiving statins. Current guidelines for the management of statin intolerance may help to guide clinical decisions, with the recommendation for patients at higher CV risk to continue statin therapy unless absolutely contraindicated [51]. While dealing with COVID-19 patients on statins, we should also take into account drug-to-drug interactions, especially with some macrolides and anti-retroviral therapy, as recently discussed in detail elsewhere [52]. 

It is recommended that those patients already on statins should continue with therapy if diagnosed with COVID-19, and adherence should be maintained to a suitable dose, according to the patient’s CVD risk. Additionally, based on the above described cholesterol reduction, plaque stabilization, CVD risk prevention, anti-inflammatory, and potential antiviral properties of statins, de novo initiation of statin therapy may be considered in high-risk patients during severe manifestations of COVID-19 to prevent some of the life-threatening cardiovascular complications.
